# Clever mothers balance time and effort in parental care: a study on free-ranging dogs

**DOI:** 10.1098/rsos.160583

**Published:** 2017-01-11

**Authors:** Manabi Paul, Shubhra Sau, Anjan K. Nandi, Anindita Bhadra

**Affiliations:** 1 Behaviour and Ecology Lab, Department of Biological Sciences, Indian Institute of Science Education and Research, Kolkata, India; 2 Department of Physical Sciences, Indian Institute of Science Education and Research, Kolkata, India

**Keywords:** free-ranging dogs, parental care, parental investment, ontogeny

## Abstract

Mammalian offspring require parental care, at least in the form of nursing during their early development. While mothers need to invest considerable time and energy in ensuring the survival of their current offspring, they also need to optimize their investment in one batch of offspring in order to ensure future reproduction and hence lifetime reproductive success. Free-ranging dogs live in small social groups, mate promiscuously and lack the cooperative breeding biology of other group-living canids. They face high early-life mortality, which in turn reduces fitness benefits of the mother from a batch of pups. We carried out a field-based study on free-ranging dogs in India to understand the nature of maternal care. Our analysis reveals that mothers reduce investment in energy-intensive active care and increase passive care as the pups grow older, thereby keeping overall levels of care more or less constant over pup age. Using the patterns of mother–pup interactions, we define the different phases of maternal care behaviour.

## 1. Background

Parental care is an essential part of mammalian development where parents, especially the mothers, invest their time, energy and resources to provide care to their offspring, enhancing the offspring's chances of survival [1,2]. Maternal care can be defined as the amount of resources invested by the mother to rear her current offspring at the cost of her own survival and future reproduction. Life-history theory predicts that the mother should invest the amount of resources for her current offspring that remains balanced against the effects on her chances of survival and future reproduction [3–5]. According to parental investment theory [6], a mother should adopt a conservative strategy that ensures her own future reproduction and survival by decreasing the allocation of resources to her current offspring [7]. This trade-off in energy allocation could be expressed through the changes in behaviours during offspring rearing [8]. Kleiman & Malcolm [9] sorted maternal care into direct and indirect care [9]. Direct maternal care comprises comparatively more energy-consuming behaviours that require direct contact between the mother and offspring [10]. In mammals, lactation seems to be the most energetically demanding component of maternal care that can affect the mother's growth, survival and reproduction [5,11,12]. Lifetime reproductive success (LRS) of the mother thus can be achieved by adjusting the intensity of direct and indirect care between parturition and weaning.

Biparental care has mostly been reported in group-living social species including humans [2,9,13,14]. Care by adults other than the parents has also been reported in cooperatively breeding species and is common although not universal in the family Canidae [10,15,16]. Cooperative breeding is well known in pack-living social canids like wolves (*Canis lupus*), coyotes (*Canis latrans*), Arctic foxes (*Vulpes lagopus*), African wild dogs (*Lycaon pictus*) and so on, where the dominant male–female pair suppresses the subordinates' reproduction [17–21]. In such species, the subordinates provide care to the offspring of the dominant pair, without reproducing themselves. In communally breeding species, however, multiple females breed at the same time and share dens or territories, and care is provided communally [22–25].

Domestic dog (*Canis familiaris*) ancestors are thought to have diverged from modern wolf (*C. lupus*) lineages 27 000 years ago [26]. Dogs are a fascinating species of carnivores displaying a diverse range of social organization, from solitary living in human homes as pets to living in social groups in free-ranging conditions [27–29]. Unlike wolves, dogs do not have any reproductive hierarchy and mate promiscuously [30]. Maternal care has been reported to be the predominant form of care received by the pups [31–33], though some amount of alloparental care is also possible, but certainly not equivalent to other members of the genus *Canis* [31,34,35]. Pet dogs are mostly supervised by their masters where pups get additional care along with parental care that ensures their better survival and development [28]. In free-ranging dogs, however, parental care is essential in the early stages of development for the survival of the pups, and in spite of the presence of parental care, the mortality rates in early life are as high as 81% [36]. Lactating females devote substantial time and energy to nurse their pups until weaning ([24], M. Paul and A. Bhadra 2016, unpublished data), thus imposing a high metabolic demand on the mothers [37]. The mother needs to provide care to ensure survival of her offspring early in their development, and she also needs to limit care in order to invest in future offspring, thereby maximizing her LRS. We carried out an extensive behavioural study to investigate the mother's investment in terms of time in parental care, and this modulates the various stages of ontogeny in the offspring.

## 2. Material and methods

In a field-based study, we collected behavioural data from 15 dog groups over a period of 15 weeks each, from the 3rd to 17th weeks of pup age. Each dog group consisted of one or more adults and pups/juveniles. A group was defined by individuals that defended a common territory and shared resources like food and shelter, prior to the denning season. All individuals in the group were uniquely named according to their coat colour and patch patterns. A group could have more than one lactating female with her current litter. We used the mother–litter units as our focal groups for behavioural observations using equal numbers of instantaneous scans and all occurrences sessions (AOS) [38]. Thus, we collected data on 22 mother–litter units (having a total of 22 mothers and 78 pups) belonging to 15 dog groups.

The study was conducted in the transit campus of IISER Kolkata, Mohanpur (22°94′ N, 88°53′ E) and Kolkata (22°34′ N, 88°24′ E), West Bengal, India, between October 2010 and February 2015, during the primary denning season [30]. As soon as a newborn litter was observed and earmarked for the study, the observer recorded their date of birth, litter size at birth and den location.

Females become very defensive just after giving birth and prevent others from entering/approaching the den [31]. The pups mostly do not emerge from the dens before the third week of age and the mother spends most of her time inside or around the dens (M. Paul 2010–2015, personal observations). Although we tried to commence observations as soon as the pups were born, we could gather very little data as many of the dens were inaccessible, and some of the mothers were very protective, leading to biased sampling. Hence we collected data equally for all the groups from the third week of pup age. Each mother–litter unit was observed for two morning (09.00–12.00) and two evening (14.00–17.00) sessions spread over blocks of two weeks. Each 3 h observation session consisted of 18 scans and 18 AOS [38], amounting to a total of 8712 scans of 1 min each and 8712 AOS of 5 min each for all the 22 mother–litter units. During each AOS, all behavioural events were recorded, with the identity of the initiator, and whenever applicable, that of the recipient. During the scans, we recorded both behavioural states and events, and used this data to estimate the time activity budgets of individuals. The AOS data were used to measure the frequency (per hour) of the observed behaviour shown by the focal individuals [38]. Any form of mother–pup interactions that could increase the chances of pup survival (such as nursing, allogrooming, regurgitation, food offering or food provisioning, den cleaning, pile sleep, pup guarding and play) were recorded as maternal care. Maternal care was sorted into active (that require direct contact with the pup, thus demanding more energy) and passive categories (not necessary to be in contact with the pup) (electronic supplementary material, S1).

### 2.1. Inter-observer variability

Two observers were involved in carrying out the behavioural observations. Before commencing the study, pilots were conducted simultaneously by the two observers on a group of dogs, and their records were tested for inter-observer variability. This was done for three dog groups, and for 10 h of data. The frequency per hour of play, nursing, allogrooming, pile sleeping, protective behaviours and foraging were compared for the two datasets using a Wilcoxon matched pairs test. As inter-observer variability was not found to be significant, the two observers continued with the study.

### 2.2. Statistics

We used StatistiXL 1.10, Statistica v. 12 and R statistics for the statistical analyses. We used ‘nlme’ package in R statistics [39] for ‘linear mixed-effects model analysis’. For the generalized linear mixed effect models, we incorporated the group identity and the year of data collection as the ‘random effects’. Predictor variables such as the pup age and current litter size (incorporating the change in litter size over time due to mortality) of the mother were added in the models as ‘fixed effects’. As the residuals play an essential part in the model validation process, we did the ‘Bartlett test of homogeneity of variances’ to check for homoscedasticity, separately for two predictor variables, i.e. age and litter size [40]. For the models where predictor variables were observed to violate the assumption of homogeneity, ‘varFixed’ weight was added to fix the model [40]. We started with the full model, i.e. with all possible two-way interactions among the fixed effects. If the two-way interaction showed no significant effect on the response variable, we reduced the model using standard protocol of backward selection method and ended up with the optimal model.

Simple linear regression was used to check the relationship between a dependent variable and a single explanatory variable. *χ*^2^-test was used to assess the goodness of fit between a set of observed and expected values (for nursing and allogrooming). ANOVAs were used to test the effect of pup age on the frequency per hour of nursing, allogrooming, protection and play behaviours shown by the mothers. We also checked for variation between the mothers (litter identities) for all these behaviours using ANOVAs. Two-sample *t*-test was used to compare the frequencies of mother- and pup-initiated play behaviour.

## 3. Results

**Figure 1.**
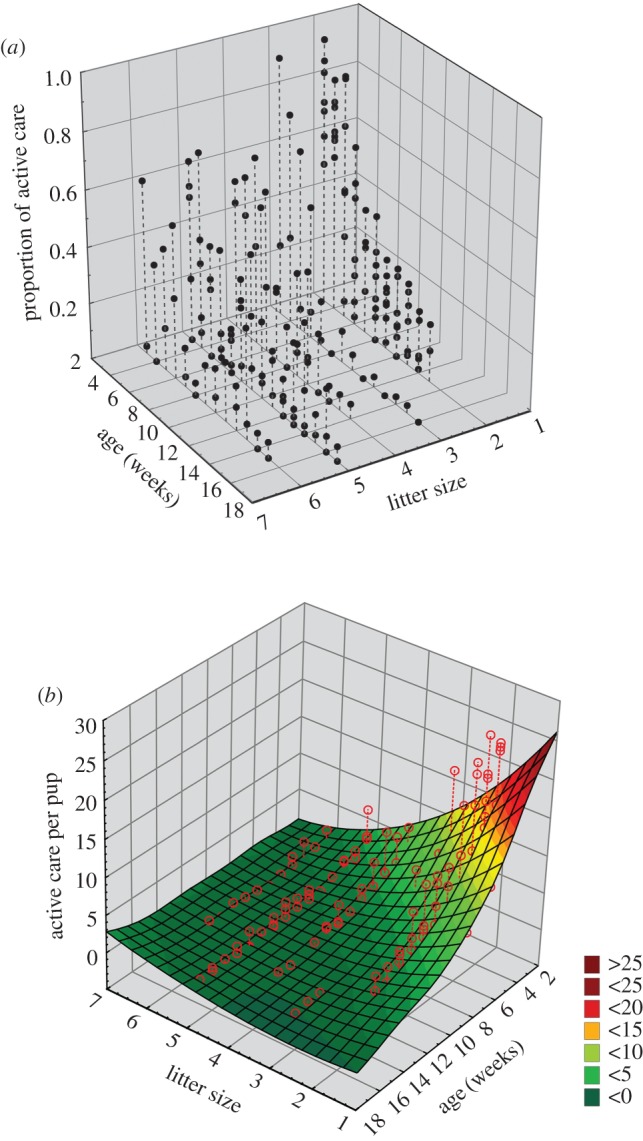
Three-dimensional graphical representation of the combined effect of pup age and current litter size on the active care shown by the mother. (*a*) Three-dimensional scatter plot showing the mother's perspective of care provided to the whole litter, at different ages, and for different litter sizes. (*b*) Three-dimensional surface plot showing the proportion of active care received by individual pups at various ages and for different litter sizes. The dark green areas depict the least and the dark red areas the maximum, levels of active care per pup.

Pup age and current litter size of the mother independently showed significant effect on the proportion of time spent by the mother in total maternal care (linear mixed effect model: *p* = age: 0.03, current litter size: 0.001) (table 1*a*; electronic supplementary material, S2). However, the time spent in active maternal care was regulated by a combination of pup age and mother's current litter size (linear mixed effect model: pup age × mother's current litter size: *p* = 4 × 10^−4^) (table 1*b*; electronic supplementary material, S3). A three-dimensional surface plot of litter size, pup age and active care revealed that the mothers having small litters showed the highest amount of active care at the early stages of pup development. However, for the same age of pups, care decreased with an increase in litter size, suggesting that the mothers regulated the investment in active care according to the litter sizes they had to nurture (figure 1*a*). Hence pups having fewer siblings tend to receive a higher amount of active maternal care at their early stages of life (linear mixed effect model: pup age × mother's current litter size: *p* < 0.0001) (table 1*c*, figure 1*b*; electronic supplementary material, S4).

**Table 1.** Results of the linear mixed effect models for the effect of pup age (age) and mother's current litter size (LS) on the proportion of time spent by the mother in maternal care, shown towards her pups. (*a*) Age and current litter size independently affect the total care shown by the mothers. (*b*) Results showing significant effect of age and litter size and the interaction between age and litter size over the proportion of time spent by the mother in active care. (*c*) Active care received by individual pup depends on the combined effect of their age and current litter size. Bold numbers represent significant values (*p* < 0.05).

	value	s.e.	d.f.	*t*-value	*p*-value
(*a*)					
(intercept)	0.9774950	0.11531726	169	8.476572	0.0000
age	−0.0218620	0.01003326	169	−2.178953	**0**.**0307**
LS	−0.0987305	0.02951282	169	−3.345342	**0**.**0010**
(*b*)					
(intercept)	1.0513085	0.09673973	169	10.867392	0.0000
age	−0.0799352	0.00898196	169	−8.899528	**0**.**0000**
LS	−0.1216618	0.02361754	169	−5.151332	**0**.**0000**
age × LS	0.0092408	0.00257809	169	3.584355	**4** × **10**^−4^
(*c*)					
(intercept)	0.5574674	0.04028909	169	13.836682	0.0000
age	−0.0377047	0.00383511	169	−9.831449	**0**.**0000**
LS	−0.0905445	0.01000378	169	−9.051032	**0**.**0000**
age × LS	0.0052365	0.00110413	169	4.923760	**0**.**0000**

**Figure 2.**
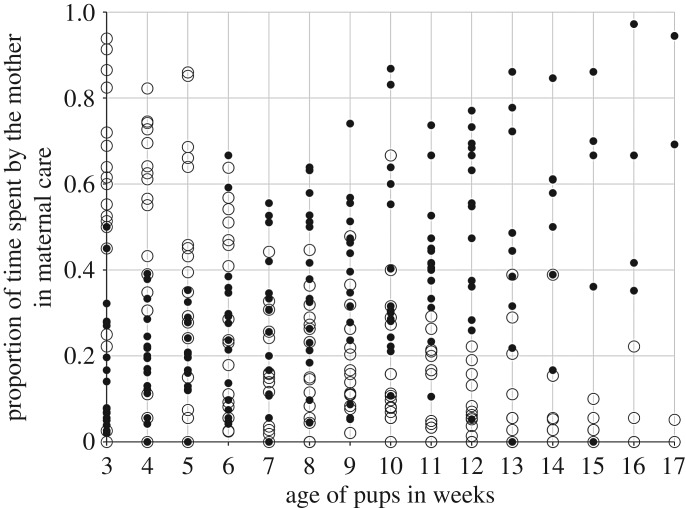
Scatter plot showing that as the pups grow older, the mothers strike a balance between the proportion of time spent in active and passive care towards their pups by decreasing the first and increasing the latter. Each dot represents a mother of a mother–litter unit. Each solid black dot represents the proportion of passive care shown by each mother, whereas each empty circle represents the proportion of active care shown by each mother.

Unlike active care, the proportion of time spent by the mother in passive care only depended on the pup age but not on the litter size (linear mixed effect model: pup age: *p* < 0.0001, current litter size: *p* = 0.85) (electronic supplementary material, S5). As the pups grew older, the mothers struck a balance between the proportion of time they spent in active and passive care towards their pups by decreasing the former and increasing the latter (linear regression comparison: *F*_1_ = 207.767, *p* < 0.0001) (figure 2).

**Figure 3.**
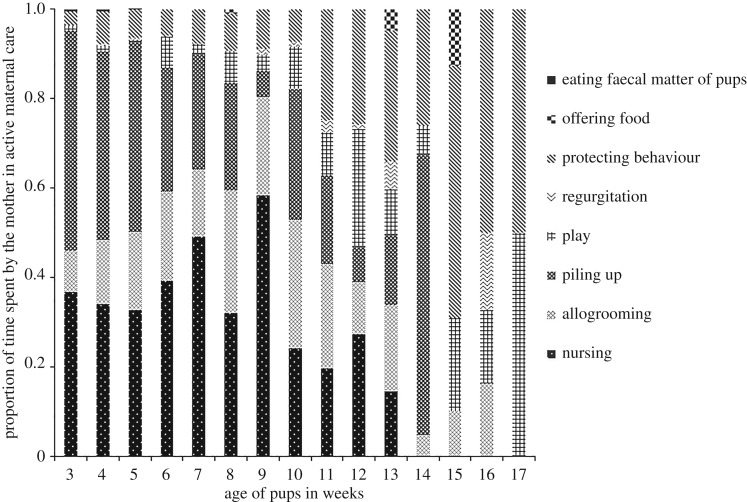
Stacked bar diagram showing that the mothers distribute their time unequally among the various active care behaviours. The mothers reduce their investment in behaviours like nursing, allogrooming and piling up with pups and invest more time in play and protective behaviours with increasing pup age.

The mother also distributed her time unequally among the various active care behaviours. With the pups growing older, the mothers reduced their investment in behaviours like nursing, allogrooming and piling up with pups (linear regression: *R*^2^ = 0.805, std. *β* = −0.897, *p* < 0.0001); and invested more time in play and protective behaviours (linear regression: *R*^2^ = 0.792, std. *β* = 0.890, *p* < 0.0001) (figure 3).

**Figure 4.**
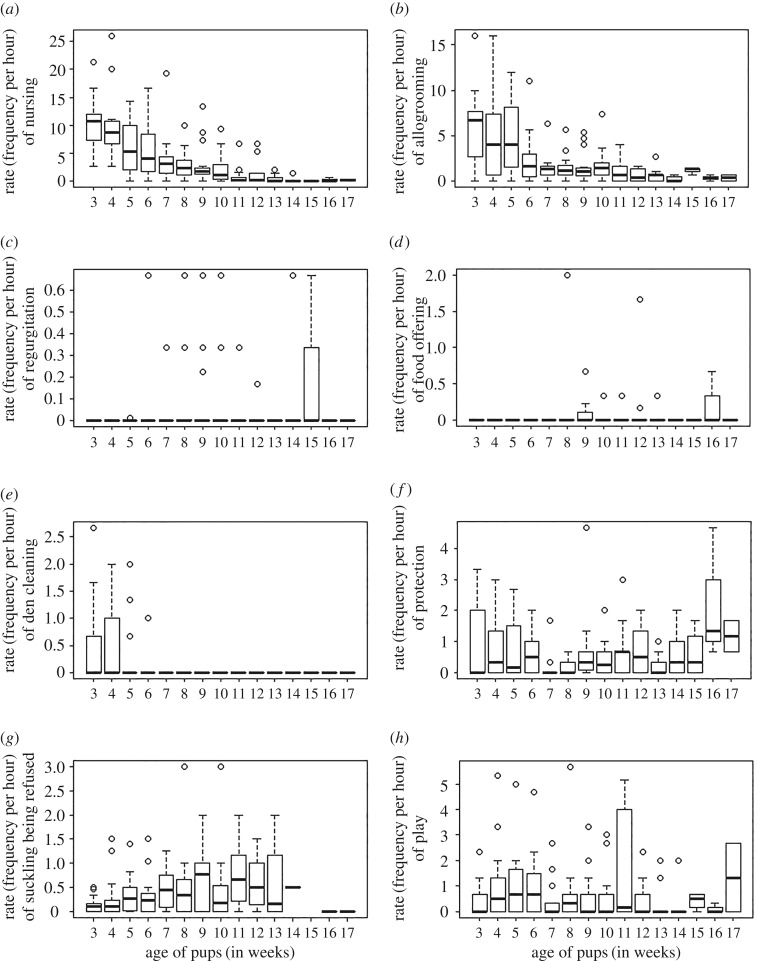
Box-whisker plots showing the rate (frequency per hour) of active care shown by the mothers towards their pups over increasing pup age. (*a*) Rate of nursing, (*b*) rate of allogrooming, (*c*) rate of regurgitation, (*d*) rate of food offering, (*e*) rate of den cleaning by eating the faecal matter of pups, (*f*) rate of protection, (*g*) rate of suckling being refused by the mother and (*h*) the rate of play interactions between mother and pups over increased pup ages.

Nursing and allogrooming occupied 33 ± 16% of the mothers' time during the third week of pup age, and reduced to 0% by the 17th week of pup age. All the 22 mothers were observed to nurse and allogroom their own pups, with a change in the rate (frequency per hour) of these behaviours over pup age (variation within mothers over pup age: ANOVA: nursing: *F* = 9.82, *p* < 0.0001; allogrooming: *F* = 6.12, *p* < 0.0001, variation between mothers: ANOVA: nursing: *F* = 1.212, *p* = 0.25; allogrooming: *F* = 1.66, *p* = 0.05, figure 4*a*,*b*). Ten of the mothers were observed to provide regurgitated food to their pups on being solicited. Regurgitation was recorded as early as the fifth week of pup age and continued until the 15th week (figure 4*c*). In eight mother–litter groups, mothers were observed to offer scavenged food to their own pups from the 9th to 16th week of pup age (figure 4*d*). Fifteen mothers were observed to eat the faecal matter of their pups in order to clean their dens until the sixth week of pup age (figure 4*e*). Behaviours like regurgitation, food offering and den cleaning, though scattered and rare in occurrence, were seen in 54.5 ± 16.4% of the total observed mother–litter units. Mothers showed aggressive behaviours such as angry barking, chasing, growling, biting and fighting to the intruder dogs and remained alert of their presence when they stayed with their pups. All the 22 mothers were observed to protect their own pups by being aggressive to the intruders, and such aggression reduced only in and around the weaning period (seventh and eighth week of pup age) (variation within mothers over pup age: ANOVA: *F* = 1.97, *p* = 0.02) (figure 4*f*). Mothers showed conflict towards their pups not by aggression but by refusing most of the pups' suckling attempts as the pups grew older (linear regression: *t* = 2.715, *p* = 0.007) (figure 4*g*). Mother–pup play interactions were recorded throughout the observation period irrespective of pup age (variation within groups over pup age: ANOVA: *F* = 1.16, *p* = 0.31) (figure 4*h*), and it was seen that pups initiated play more frequently than their mothers (two-sample *t* test: *t* = −4.402, *p* < 0.0001); 73% of the total observed mother–pup play behaviours being pup-initiated.

**Figure 5.**
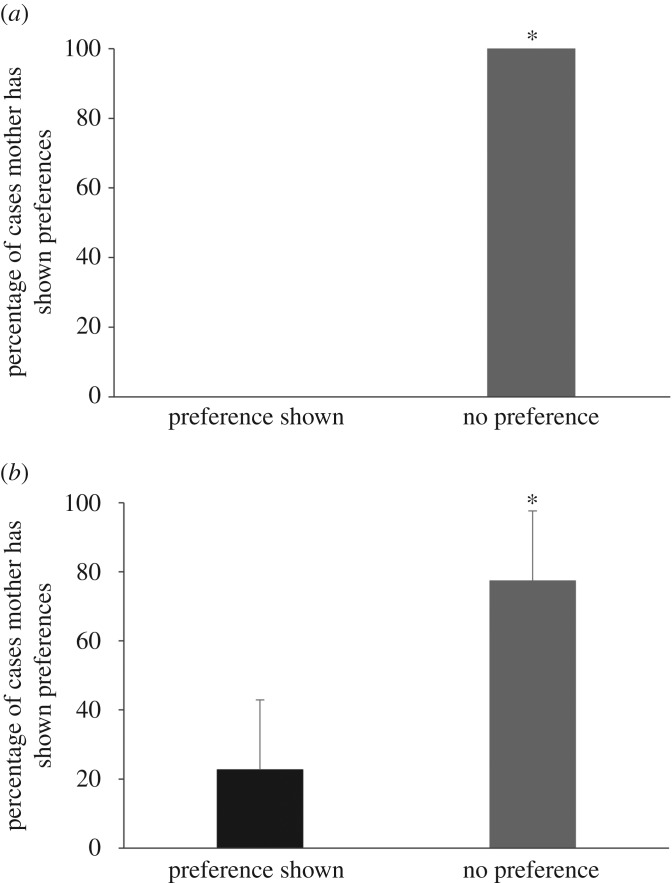
Bar diagram showing (*a*) the preference shown by the mother while she nurses her pups and (*b*) the preference shown by the mother while she allogrooms her pups. Asterisk indicates statistically significant difference (*p* < 0.05).

The mothers did not show preferential treatment towards any individual pups in nursing initiation (*χ*^2^-test: *χ*^2^ = 100, *p* < 0.0001, figure 5*a*) and allogrooming (*χ*^2^-test: *χ*^2^ = 29.160, *p* < 0.0001, figure 5*b*) during the entire duration of observations, and thus can be considered to be quite impartial. However, the rate (frequency per hour) of care received (in terms of nursing and allogrooming) by individual pups was regulated by a combination of pup age and the current litter size (linear mixed effect model: pup age × current litter size: *p* = 0.00124) (table 2; electronic supplementary material, S6).

**Table 2.** Results of the linear mixed-effects model for the rate of care received by individual pup (in terms of nursing and allogrooming). The rate of care received is regulated by a combination of pup age and their current litter size. Bold numbers represent significant values (*p* < 0.05).

	value	s.e.	d.f.	*t*-value	*p*–value
(intercept)	9.61189	0.91536	169	10.501	2.22 × 10^−16^
age	−0.73337	0.08936	169	−8.207	**6.66** × 10**^−14^**
LS	−1.12415	0.23868	169	−4.710	**7.24** × 10**^−6^**
age × LS	0.08342	0.02542	169	3.282	**0.00124**

### 3.1. Phases of maternal care

It has been suggested that there are critical periods during the ontogeny of pups, which affect the development of social behaviour [41]. As parental care contributes significantly to development in early life, we used the changing pattern of the various maternal care behaviours to draw up a timeline of pup development. Nursing and allogrooming became less frequent from the seventh week of pup age, with most suckling attempts by pups being refused by the mother from this stage. Hence the seventh week of pup age marks the onset of weaning. Mothers also stop den cleaning by eating their pups' faecal matter from the seventh week of pup age. However, mothers seem to prepare for the process of weaning much earlier, i.e. the fifth week of pup age, by providing regurgitated food to the pups, thereby supplementing nursing and initiating the pups to scavenged food. All these care-giving behaviours stop at the 16th week of pup age, demarcating the time when the pups become independent of the mothers (figure 4). Based on our analysis, we divide the developmental phase of a dog's life into two stages. The pups receive active maternal care at high rates up to the sixth week of age, and from the 7th to 13th week of age, we see the weaning period, when pups gradually become less dependent on the mothers. By the 16th week of age, they are completely independent of the mother. Thus, the 13th week demarcates the beginning of the juvenile phase of the dog's life, which is also a period of active learning and cognitive development.

## 4. Discussion

Free-ranging dogs are known to have a facultatively social structure that is mostly influenced by their mating and pup-rearing needs [30,42,43]. During the denning season, social groups may contain more than one lactating female and her current litter, in addition to other adults of the group, both male and female. Adult group members other than the mother sometimes contribute to pup rearing, but at a level lower than the care provided by the mother (M. Paul and A. Bhadra 2016, unpublished data, [34]). In mammals, maternal care involves mother–offspring behavioural interactions that enhance the mother's reproductive success by ensuring the offspring's survival [44]. Disruption of maternal care often leads to adverse effects on the developing young [44]. In this study, maternal care was observed to be present in all the observed litters, and showed consistent patterns over pup age and litter size, with maximum care being provided at the earliest stages of pup development. Despite inter-individual variation, there was a consistent trend in higher care being provided per pup when litter sizes were small, leading to pups with fewer siblings receiving more care in their early life.

Mothers invested a substantial amount of time in active care like nursing, allogrooming, piling up and so on, at the very early stages of pup development, but adjusted their time activity budget to replace active care with passive care as the pups grew older. Nursing and allogrooming are both energy-intensive behaviours [37,45], and reducing investment in such behaviours can help the mother to replenish her energy reserves for future reproduction [7,8]. A sharp decline in the rates of nursing and allogrooming were accompanied by offering of regurgitated and scavenged food to the pups in the pre-weaning period. Hence, while the mothers tried to wean the pups from milk, they also provisioned them with solid and semi-solid food. Unlike adults, pups in the weaning stage do not show a strong preference for protein-rich food. It is possible that they learn to selectively scavenge on protein-rich food through the food provisioned by the mother, suggesting the possibility of a process of training [46]. Pile sleep and piling up, i.e. grouping of mother and pups in a den, has also been observed to be an important maternal care behaviour that helps to maintain the body temperature of newborn pups [47]. Such a close attachment between mother and pups was observed in the free-ranging dogs in the first couple of weeks of observation, and decreased as the pups grew older. Such close interactions are likely to have been even higher during the first two weeks after pup birth, when the mothers spent most of their time inside the dens. While these close contact behaviours reduced, play and protection increased with pup age, once again showing a switch from active to passive parental care.

We report three distinct phases in the ontogeny of free-ranging dogs, which is manifested through the changing maternal care patterns. Up to the sixth week of age, the pups receive ample active maternal care, and after this stage the onset of weaning occurs. The 7th to 13th week of age demarcates the ‘zone of conflict’ over weaning between the mother and her pups ([42,43]; M. Paul and A. Bhadra 2016, unpublished data). We demarcate the 13th week as the end of the ‘pup’ stage of life and the beginning of the ‘juvenile’ phase. The juvenile phase continues beyond the post-weaning phase, when the mother–offspring interactions are more of social than physiological significance. By the 16th week of age, maternal care of all kind ceases, though the juveniles continue to share space and resources with the mother [27]. This phase lasts till the onset of sexual maturity and eventual adulthood.

In mammals, age-specific decline in female fertility followed by a sudden collapse in reproduction has been reported in various animals including dogs [48]. Free-ranging dogs in India face high early-life mortality of nearly 81%, leading to reduced fitness for the mothers. According to the predictions of life-history theory [3–5], mothers could be expected to strike a balance between the cost and benefit of reproduction. Thus, the investment in one batch of offspring in terms of time and energy devoted to parental care (cost) should be adjusted to maximize their LRS (benefit) [7]. Our study reveals that free-ranging dog mothers maximize their opportunity for future investment in reproduction by adjusting the active–passive care ratio over pup age, gradually shifting from more energy-intensive care to less energy-intensive care. This enables the mothers to provide prolonged care to their offspring at a relatively low investment. Humans often carry away pups and kill them, sometimes accidentally but at times on purpose [36]. The prolonged attachment with the mother might help to increase their fitness by providing protection to pups especially from interfering humans and by helping pups to learn efficient foraging strategies from their mothers through cultural transmission.

## Supplementary Material

ESM1: Ethogram of mother-pup interactions ESM2: Details of the linear mixed effect model that shows the effect of pup age and their current littersize on the proportion of time spent in total care by the mother. ESM 3: Details of the linear mixed effect model that shows the effect of pup age and their current littersize on the proportion of time spent in active care by the mother. ESM 4: Details of the linear mixed effect model that shows the effect of pup age and their current litter size on the active care received per pup. ESM5: Details of the linear mixed effect model that shows the effect of pup age and their current littersize on the proportion of time spent in passive care by the mother. ESM6: Details of the linear mixed effect model that shows the effect of pup age and their current littersize on rate of care received by individual pups.

## Data accessibility

The complete dataset for this manuscript can be accessed at the Dryad Digital Repository: http://dx.doi.org/10.5061/dryad.d7v27 [49].
