# Supplementary material for: Clever mothers balance time and effort in parental care: a study on free-ranging dogs
Source: R Soc Open Sci. 2017 Jan 11;4(1):160583. doi: 10.1098/rsos.160583 (PMC5319321; doi:10.1098/rsos.160583)
Supplement: ESM1: Ethogram of mother-pup interactions ESM2: Details of the linear mixed effect model that shows the effect of pup age and their current littersize on the proportion of time spent in total care by the mother. ESM 3: Details of the linear mixed effect model that shows the effect of pup age and the [file rsos160583supp1.pdf]

Clever mothers balance time and effort in parental care: a study on free-ranging dogs

Manabi Paul[1], Shubhra Sau[1], Anjan K. Nandi[2] and Anindita Bhadra[1*]

[1] Behaviour and Ecology Lab, Department of Biological Sciences,

Indian Institute of Science Education and Research – Kolkata, India

[2] Department of Physical Sciences,

Indian Institute of Science Education and Research – Kolkata, India

[*]**Address for Correspondence:**

Behaviour and Ecology Lab, Department of Biological Sciences,

Indian Institute of Science Education and Research – Kolkata

Mohanpur Campus, Mohanpur,

PIN 741246, West Bengal, INDIA

tel. 91-33-66340000 ext 1223

e-mail: abhadra@iiserkol.ac.in

Active maternal care		
**Code**	**Behaviour**	**Description of the behaviour**
AG	Allo groom	To clean the coat of their pups by licking, to remove dirt and parasites.
CK	Nursing	To lie down or stand still in order to allow pups to suckle (feed on breast milk).
EF	Eat fecal matter	Mother eats fecal matter of her pups to clean her den.
OF	Offer food	Mother brings food obtained through scavenging/ begging in her mouth and drops it on the ground in the vicinity of the pups, allowing them to eat.
PL	Play	Individuals engage in affiliative activities like bowing down, tail wagging, mock biting, jumping, tumbling, etc. for enjoyment.
PS	Pile sleep	Individuals pile up together while sleeping ensuring maximum body contact.
PU	Pile Up	Individuals pile up together while resting ensuring maximum body contact.
TH	Threat	Individuals assume an aggressive posture with raised tail and ears, exposed jaws, fore legs stretched forward, producing a deep throated growl and staring at the recipient for a prolonged time.
VM	Vomit	Expels the semi digested and semi solid contents of stomach by regurgitation.
**Passive maternal care**		
**Code**	**Behaviour**	**Description of the behaviour**

DW	Drink water	Individuals drinking water from sources like puddles, shallow drains, dripping taps, etc.
ET	Eating food	Individuals feeding on any solid or semi-solid food items.
FS	Food search	Individuals wander around and search thoroughly (by using the visual, olfactory or tactile cues) for food*.
GR	Groom	Individuals clean their own bodies by licking, to remove dirt and parasites.
LG	Scratching by legs	Individuals use their claws while sitting or standing, to scratch themselves. This is a self-maintenance behaviour.
LI	Licking	Individuals pass their tongue over their body to make it clean.
OT	Resting/ standing/ lazing	Individuals sit, stand or lie down and remain immobile for several (at least 2) minutes at a stretch.

ESM 1. A table showing the ethogram for maternal care. A unique two letter code was used for recording each behaviour during observations. The table shows the code, the name of the behaviour and its description. Maternal care was divided into active and passive care. Active care behaviours involved direct social interactions between the mother and pups. The passive care behaviours were not interactions, but individual actions of the mother occurring in the vicinity of the pups which allowed them to share time and space with and provide protection to the pups. In case of social interactions, the individual starting the interaction was designated as the initiator and the one towards which the behaviour was shown, was the recipient (Please see Martin and Bateson 2007 *Measuring Behaviour: An Introductory Guide*, 3rd edn. Cambridge University Press).

(*) see Bhadra et al. 2016 for different types of food eaten by free-ranging dogs.

ESM 2

Details of the linear mixed effect model that shows the effect of pup age and their current litter size on the proportion of time spent in total care by the mother.

In order to check the effect of both the predictor variables i.e. pup age and mother's current litter size, we ran a "linear mixed effect model" incorporating the predictor variables as the "fixed effects" whereas the proportion of time spent by the mother in total care was included in the model as the "response variable". We collected the data on maternal care from 15 different dog groups that had 22 mother-litter units, over a span of 5 years (2010-2015). Hence the identity of each mother-litter units (fgr) and the year of data collections (fyr) were incorporated in the model as the "random effects". A Gaussian distribution was considered for the response variable in the model. We started with the full model (mod1), i.e., with all possible two-way interactions among the fixed effects.

Variables used in the model:

Response variable:

Proportion of time spent by the mother in total care- **totpcprop**

Fixed effects:

Age of pups in weeks- **age**

Current litter size- **LS**

Random effects:

Mother-litter unit's identity- **fgr**

Year of observation- **fyr**

Model: **mod1<- lme (totpcprop~ age*LS, random = ~1|fgr/fyr)**

Results

	Value	Std. Error	DF	t-value	p-value

(Intercept)	0.9774950	0.11531726	169	8.476572	0.0000
age	-0.0218620	0.01003326	169	-2.178953	**0.0307**
LS	-0.0987305	0.02951282	169	-3.345342	**0.0010**
age*LS	0.0037576	0.00284882	169	1.318992	0.1890

The two-way interaction shown no significant effect on the response variable and hence we reduced the model using standard protocol of backward selection method and ended up with the optimal model (mod2).

Model: **mod2<- lme (totpcprop~ age + LS, random = ~1|fgr/fyr)**

Results

	Value	Std. Error	DF	t-value	p-value
(Intercept)	0.9774950	0.11531726	169	8.476572	0.0000
age	-0.0218620	0.01003326	169	-2.178953	**0.0307**
LS	-0.0987305	0.02951282	169	-3.345342	**0.0010**

ESM 3

Details of the linear mixed effect model that shows the effect of pup age and their current litter size on the proportion of time spent in active care by the mother.

The predictor variables i.e. the “age” and “LS” were incorporated in the generalized linear mixed effect model to check their effect on the response variable i.e. the proportion of time spent in active care (acareprop) for 22 mother-litter units, collected over a span of 5 years. Identity of each mother-litter unit (fgr) and the year of data collection (fyr) were added in the model as the “random effects”. We started with the full model (mod3), i.e., with all possible two-way interactions among the fixed effects.

Model validation

Since the residuals have an essential role in the model validation process, we did the “Bartlett test of homogeneity” of variances to check the presence of homoscedasticity in the model, separately for two predictor variables i.e. pup age (age) and current litter size (LS). P-value for the predictor “age” exhibited violation in the homogeneity assumption and it was fixed by adding “varFixed” as the “weight” in the model.

Variables used in the model:

Response variable:

Proportion of time spent by the mother in active care- **acareprop**

Fixed effects:

Age of pups in weeks- **age**

Current litter size- **LS**

Random effects:

Mother-litter unit’s identity - **fgr**

Year of observation- **fyr**

Model: **mod3<- lme(acareprop ~ age * LS, random= ~1|fgr/fyr, weights= varFixed(~age))**

Results

	Value	Std. Error	DF	t-value	p-value
(Intercept)	1.0513085	0.09673973	169	10.867392	0.0000
age	-0.0799352	0.00898196	169	-8.899528	0.0000
LS	-0.1216618	0.02361754	169	-5.151332	0.0000
age*LS	0.0092408	0.00257809	169	3.584355	**4e-04**

Here the two-way interaction between the age and LS showed significant effect on the response variable and hence we retained this model.

ESM 4

Details of the linear mixed effect model that shows the effect of pup age and their current litter size on the active care received per pup.

Total active care shown by the mother for a particular litter was divided equally among the pups that were observed to be present for the respective week of pup age. Active care received per pup was incorporated into the generalized linear mixed effect model as the response variable. We wanted to check the effect of predictor age and LS on the amount of active care received per pup. Identity of each mother-litter unit (fgr) and the year of data collection (fyr) were added in the model as the “random effects”. We started with the full model (mod4), i.e., with all possible two-way interactions among the fixed effects.

Model validation

Since the residuals have an essential role in the model validation process, we did the “Bartlett test of homogeneity” of variances to check the presence of homoscedasticity in the model, separately for two predictor variables i.e. pup age (age) and current litter size (LS). P-value for the predictor “age” exhibited violation in the homogeneity assumption and it was fixed by adding “varFixed” as the “weight” in the model.

Variables used in the model:

Response variable:

Active care received per pup- **acareperpup**

Fixed effects:

Age of pups in weeks- **age**

Current litter size- **LS**

Random effects:

Mother-litter unit’s identity- **fgr**

Year of observation- **fyr**

Model: **mod4<- lme(acareperpup ~ age * LS, random= ~1|fgr/fyr, weights= varFixed(~age))**

Results

	Value	Std. Error	DF	t-value	p-value
(Intercept)	0.5574674	0.04028909	169	13.836682	0.0000
age	-0.0377047	0.00383511	169	-9.831449	**0.0000**
LS	-0.0905445	0.01000378	169	-9.051032	**0.0000**
age*LS	0.0052365	0.00110413	169	4.923760	**0.0000**

Here the two-way interaction between the age and LS showed significant effect on the response variable and hence we retained this model.

ESM 5

Details of the linear mixed effect model that shows the effect of pup age and their current litter size on the proportion of time spent in passive care by the mother.

The predictor variables i.e. the "age" and "LS" were incorporated in the generalized linear mixed effect model to check their effect on the response variable i.e. the proportion of time spent in passive care (pcareprop) for 22 mother-litter units, collected over a span of 5 years. Identity of each mother-litter unit (fgr) and the year of data collection (fyr) were added in the model as the "random effects". We started with the full model (mod5), i.e., with all possible two-way interactions among the fixed effects.

Variables used in the model:

Response variable:

Proportion of time spent by the mother in passive care- **pcareprop**

Fixed effects:

Age of pups in weeks- **age**

Current litter size- **LS**

Random effects:

Mother-litter unit's identity - **fgr**

Year of observation- **fyr**

Model: **mod5<- lme(pcareprop ~ age * LS, random= ~1|fgr/fyr, weights= varFixed(~age))**

Results

	**Value**	**Std. Error**	**DF**	**t-value**	**p-value**
(Intercept)	0.05735617	0.08746429	169	0.655767	0.5129
age	0.04428560	0.00786539	169	5.630439	0.0000

LS	0.00443542	0.02273035	169	0.195132	0.8455
age*LS	-0.00370192	0.00225732	169	-1.639963	0.1029

The two-way interaction showed no significant effect on the response variable and hence we reduced the model using standard protocol of backward selection method and ended up with the optimal model (mod6).

Model: **mod6<- lme(pcareprop ~ age + LS, random= ~1|fgr/fyr, weights= varFixed(~age))**

Results

	**Value**	**Std. Error**	**DF**	**t-value**	**p-value**
(Intercept)	0.05735617	0.08746429	169	0.655767	0.5129
age	0.04428560	0.00786539	169	5.630439	**0.0000**
LS	0.00443542	0.02273035	169	0.195132	0.8455

ESM 6

Details of the linear mixed effect model that shows the effect of pup age and their current litter size on rate (frequency per hour) of care received (in terms of suckling and allogrooming) by individual pups.

The predictor variables i.e. the "age" and "LS" were incorporated in the generalized linear mixed effect model to check their effect on the response variable i.e. the rate of (frequency per hour) of care received (in terms of suckling and allogrooming) by individual pups (carercvd) for 22 mother-litter units, collected over a span of 5 years. Identity of each mother-litter unit (fgr) and the year of data collection (fyr) were added in the model as the "random effects". We started with the full model (mod7), i.e., with all possible two-way interactions among the fixed effects.

Variables used in the model:

<u>Response variable:</u>

The rate of (frequency per hour) of care received (in terms of suckling and allogrooming) by individual pups - **carercvd**

<u>Fixed effects:</u>

Age of pups in weeks- **age**

Current litter size- **LS**

<u>Random effects:</u>

Mother-litter unit's identity - **fgr**

Year of observation- **fyr**

Model: **mod7<- lme(carercvd ~ age * LS, random= ~1|fgr/fyr)**

Results

	Value	Std. Error	DF	t-value	p-value
(Intercept)	9.61189	0.91536	169	10.501	2.22e-16
age	-0.73337	0.08936	169	-8.207	**6.66e-14**
LS	-1.12415	0.23868	169	-4.710	**7.24e-06**
age*LS	0.08342	0.02542	169	3.282	**0.00124**
